# Spermatophore development in drones indicates the metabolite support for sperm storage in honey bees (*Apis cerana*)

**DOI:** 10.3389/fphys.2023.1107660

**Published:** 2023-02-22

**Authors:** Lele Yan, Huali Song, Xiangyou Tang, Xiaomei Peng, Yaohui Li, Huan Yang, Zeyang Zhou, Jinshan Xu

**Affiliations:** ^1^ College of Life Sciences, Chongqing Normal University, Chongqing, China; ^2^ Key Laboratory of Conservation and Utilization of Pollinator Insect of the upper reaches of the Yangtze River, Ministry of Agriculture and Rural Affairs, Chongqing, China

**Keywords:** *Apis cerana*, drones, spermatophore, squalene, sperm viability

## Abstract

Developing effective long-term sperm storage strategies to maintain activity requires an understanding of the underlying spermatophore developmental phase in drones. Here we compared the developmental processes and metabolites about seminal vesicles of drones from different parentages (0-24 d)in honeybee colonies, including mated queens, virgin queens, and worker bees. The results showed a similar developmental trend of seminal vesicles in thethree groups of drones on the whole, although there were significant differences in developmental levels, as well as in other indicators. Correlation analysis showed significant positive correlations between seminal vesicle width and sperm viability. The metabolomics of the seminal vesicles in drones from mated queens showed differences of the metabolites in each stage. Particularly, squalene identified among them was validated a protective effect on sperm vitality *in vitro* experiments. Together the results of these assays support that there were significant differences in the developmental levels of seminal vesicles among the three groups of drones in honeybees, wherein a significant correlation between sperm viability and the developmental levels of seminal vesicles were dissected. The metabolomics analysis and semen storage experiments *in vitro* display signatures of squalene that may act as an effective protective agent in maintaining sperm viability. Collectively, our findings indicate that spermatophore development in drones provides metabolite support, which contributes to research on the differences of sperm viability among drones in the future.

## 1 Introduction

As the typical model insect, honey bees were used to explore haploid and diploid characteristics (Palmer and Oldroyd, 2000; Sumner et al.,2018). Worker bees and queens developed from diploid fertilized eggs, in contrast to drones, which developed from haploid unfertilized eggs in colonies (Stürup et al.,2013). To mate with the queen during the spring and autumn breeding seasons is the main function of drones, to increase the population of colonies (Winston, 1991). The reproductive potential of both the mated queens and drones generally determined the quality of colonies. In recent studies, they had shown an average of 12–14 drones would mate with the virgin queens during a single nuptial flight (Estoup et al.,1995; Tarpy et al., 2000; Rhodes, 2002; Ben Abdelkader et al., 2014). Therefore, the reproductive potentials of drones were directly related to the reproductive health of the queen, as well as the stability of the colony. The reproductive potential was closely related to the indicators of drones, including body size and sperm viability, Compared to other drones, small drones have a reproductive disadvantage, for example in the amount of sperm, and size of spermatophore (Berg et al., 1997). The wing length of small drones, which emerged from worker cells, was found to be about 13% smaller than the wings of large drones which emerged from drone cells in *Apis mellifera*, and there was a significant positive correlation between body size and sperm number (Schlüns et al., 2003). The study has shown that drones from mated queens were more successful than drones from worker bees in sperm competition (Gençer and Kahya, 2020).

Sperm transferred from the testes to the seminal vesicles was completed in the week after plumage, which signaled the beginning of sexual maturity in drones (Snodgrass, 1956). The development of the seminal vesicles was closely related to sperm viability, which was the main tissue for drones to store sperm after plumage. The developmental process of the drones has been completely described in *Apis mellifera*, especially since there was a significant positive correlation between the seminal vesicle length and sperm viability (Metz and Tarpy, 2019). However, both the reproductive developmental processes of different drones and the metabolites of seminal vesicles at different developmental stages have been poorly studied in honey bees. It was important to analyze the reproductive development of different drones, as well as the metabolites of the seminal vesicles at different developmental stages in honey bees, which would help us to understand more clearly the reasons for reproductive differences and changes in sperm viability of different drones.

In this study, we compared the seminal vesicles of three groups of drones from different parentages, including mated queens (MQD), virgin queens (VQD), and worker bees (WBD) in honeybee colonies. For understanding the differences in metabolites of the seminal vesicles at different developmental stages, GC-MS was used to analyze the seminal vesicles of drones from the mated queens (MQD) at different developmental stages. In general, it set the basis for exploring the mechanisms of long-term sperm storage and provided a new direction for studying the differences in sperm viability between different drones.

## 2 Materials and methods

### 2.1 Source of sample

The experimental colonies were obtained from the Genetic Base of Honey Bee Germplasm Resources of Chongqing Normal University. They were fed in March and April to promote early spring reproduction. For the queen-rearing treatment, a strong colony was selected as the maternal colony to choose eggs for hatching the virgin queens larvaes. Then, nine colonies numbered in order from 1 to 9, were selected as feeding colonies for subsequent treatments. The virgin queens of colonies 1 to 3 were mated naturally; the virgin queens of colonies 4 to 6 were anesthetized with CO_2_ (concentration 70% for 7 min) at 5 days and 6 days respectively. Lastly, colonies 7 to 9 were used to promote egg-laying by worker bees. Keep all colonies well-fed with nectar.

### 2.2 Drones collection and indicators measurement

The capped drones were placed in an indoor incubator 24 h before they emerged. Drones plumaged that day were marked for 0 d, the next day for 1 d, and so on. All marked drones were returned to the original colonies for rearing. For subsequent experimental analysis, drones were collected at an even number of ages from 0 to 24 days. Samples were collected from the corresponding colonies at the appropriate age and measured their indicators, including body mass, body length, thorax width, right forewing length, as well as the mucous glands and the seminal vesicles. Please see the Supplementary Figure S1 for the measurement indicators (Supplementary Figure S1). Finally, the seminal vesicles were placed in sterile centrifuge tubes for subsequent experimental processing. There were 351 drones were dissected and measured in our study.

### 2.3 Sperm viability analysis

Seminal vesicles were placed in a buffer (The buffer consisted of 3.6% trisodium citrate dihydrate, 0.32% sodium bicarbonate, 0.06% potassium chloride, and 0.5% glucose, leveled by distilled water.) for fragmentation. The Hoechst 33342 and PI double staining methods were used to perform live and dead cell counts with a hemocytometer plate. The nuclei of living cells were stained blue by Hoechst 33342, while dead nuclei were stained red by PI. The sperm count and viability were obtained by counting the blue and red nuclei (Supplementary Figure S2). We added drops of semen to cover the entire counting area of the hemocytometer plate. The sperm in the upper left, bottom left, upper right, bottom right, and middle areas of the counting areas were photographed and counted in a 200x field of view and calculated according to the 25 × 16 hematology plate formula. The number of live cells was defined as sperm viability in our study. Sperm were counted by reference to the following formula of 25 × 16 hematology plate:

$$T=\left(L1+L2+R1+R2+M\right)/80\times 400\times 10000\times D$$

where T is total count, L1 is the number of upper left, L2 is the number of bottom left, R1 is the number of upper right, R2 in the number of bottom right, M is the number of middle areas and D is the dilution rate.

### 2.4 Gas chromatography-mass spectrometry identification

The seminal vesicles were extracted with methanol before being analyzed by GC-MS. The GC-MS conditions were as follows: DB-5 capillary column, helium carrier gas with purity 99.999%, ion source temperature 250°C, inlet temperature 300°C, interface temperature 100°C, injection split ratio 20:1, injection volume 1 μL, oven temperature program: initial temperature 100°C for 1 min, then increasing to 250°C at 4°C/min, maintaining at 250°C for 3 min, scan rang 50–550 m/z with an even time 0.2 s and solvent delay time 10 min.

### 2.5 *In vitro* experiments

Squalene was purchased from Shanghai Macklin Biochemical Technology Co. 10 ul of semen and 10 ul of different concentrations of squalene were added to the three experimental groups, and the buffer was added to the control group. The final concentrations of the solutions were 0.5 mol/L and 1 mol/L respectively, and all groups were placed at 4°C. Sperm viability was analyzed at 0, 12, 24, and 36 h respectively.

### 2.6 Data analysis

Images were processed using Image. At the same magnification and camera, each image was calibrated to 1.00 mm and measured using toupview software. One-way ANOVA and multiple comparisons were used to test for significant differences in IBM SPSS Statistics 21 with GraphPad Prism 8.0.2.

## 3 Results

### 3.1 Morphological analysis

**FIGURE 1:**
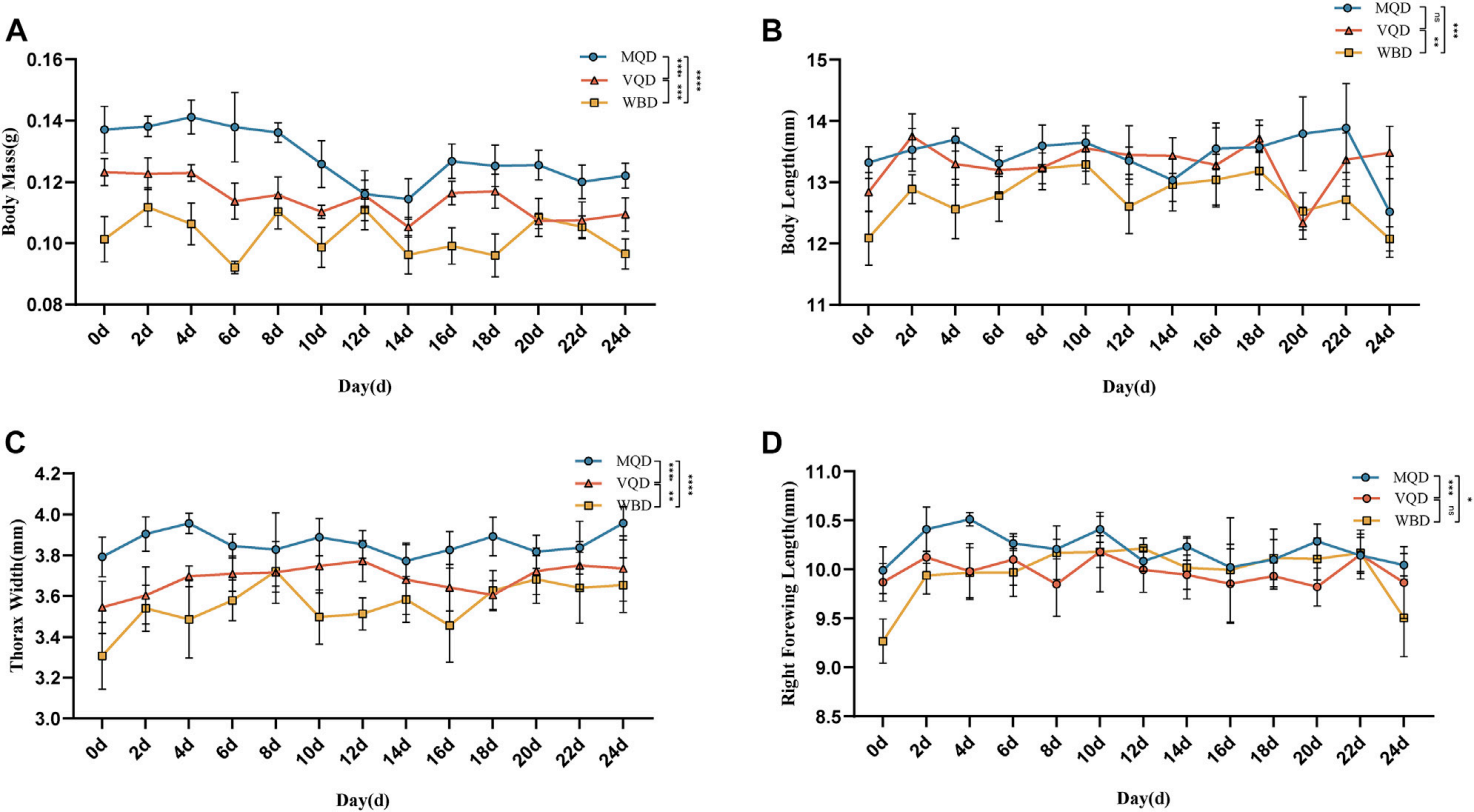
Morphological indicators of three groups of drones from 0 to 24 days: **(A)** body mass; **(B)** body length; **(C)** thorax width; **(D)** right forewing length; MQD: drones from mated queens; VQD: drones from virgin queens; WBD: drones from worker bees.

Morphological measurements and data analysis of three groups of drones from 0 to 24 days showed a decreasing trend in body mass (Figure 1A).

There was a rapid developmental phase of body length from 0 to 2 days (Figure 1B). There was no significant difference between MQD and VQD throughout their development (*p* > 0.05), while WBD was significantly different from the other two drones (*p* < 0.05). All three groups of drones showed a trend toward a slow decrease in body length (Figure 1B). There was a significant developmental difference in thorax width in the three groups of drones (*p* < 0.05). There was a clear developmental trend in MQD and VQD from 0–4 d, while in WBD it was from 0–8 d. It showed a greater trend in WBD throughout development (Figure 1C). With increasing age, the right forewing length showed a slow decrease (Figure 1D). There was a significant difference between MQD and the other two drones throughout development (*p* < 0.05), while no significant difference was found between VQD and WBD (*p* > 0.05). There were significant differences (*p* < 0.05) among the three groups of drones in morphological indicators throughout the developmental processes, including body mass, body length, thorax width, and right forewing length.

### 3.2 Comparison of gonads

**FIGURE 2:**
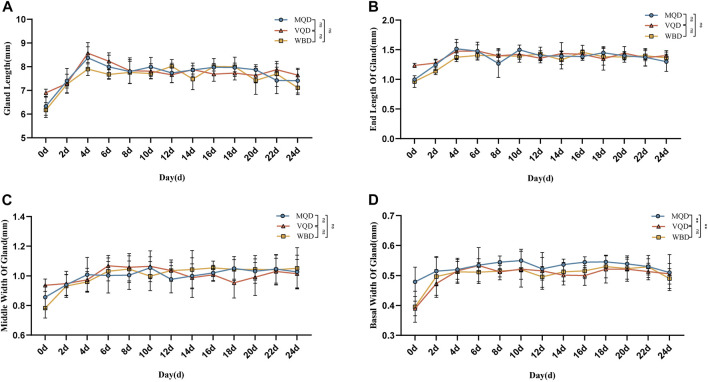
Age-related development processes of mucous glands in drones:**(A)** length of the mucous gland; **(B)** end width of the mucous gland; **(C)** middle width of the mucous gland; **(D)** basal width of mucous gland MQD: drones from mated queens; VQD: drones from virgin queens; WBD: drones from worker bees.

**FIGURE 3:**
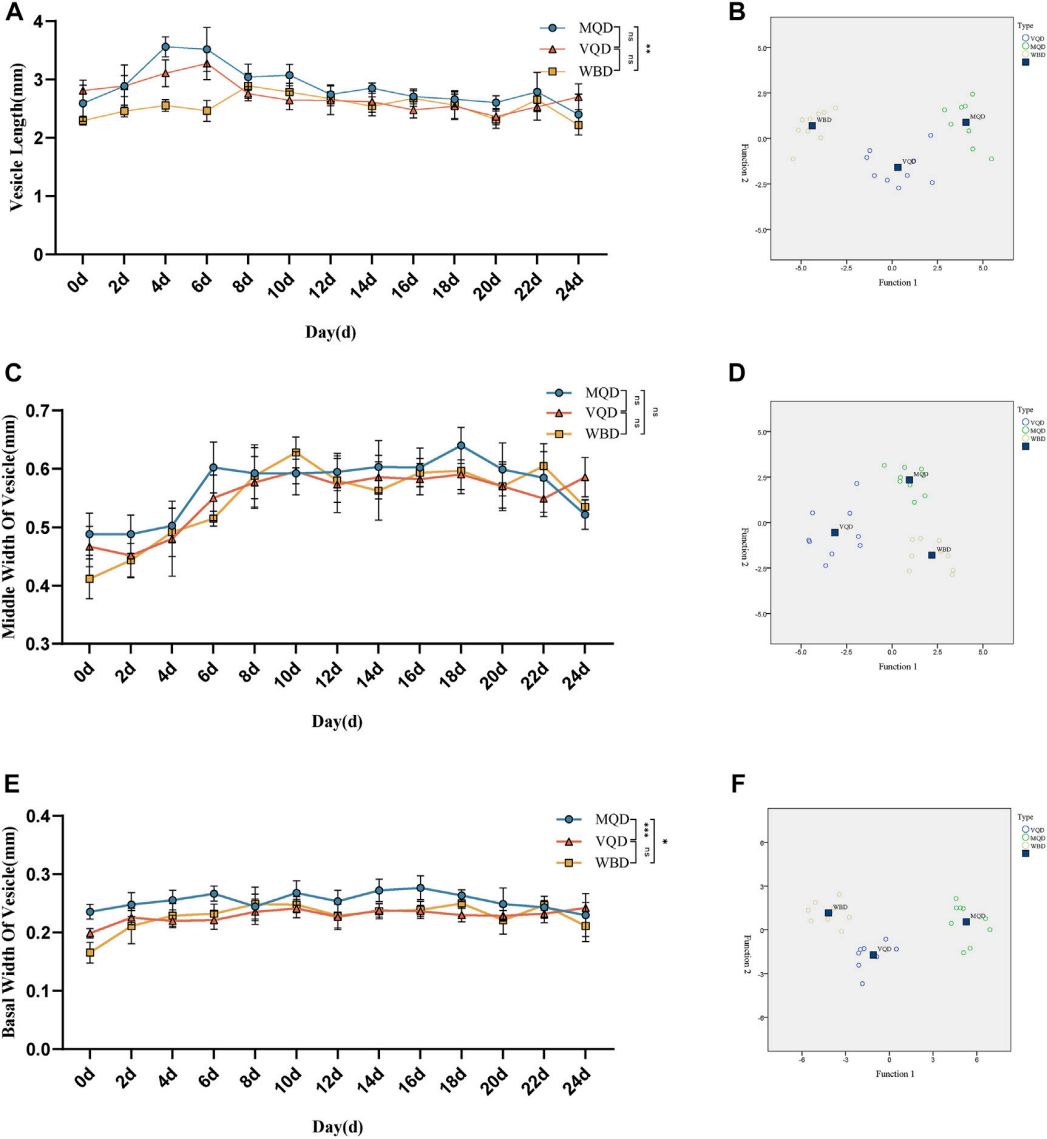
Age-related development processes of seminal vesicles in drones: **(A)**length of seminal vesicles; **(B)** discriminant analysis of length; **(C)** middle width of seminal vesicles; **(D)** discriminant analysis of middle width **(E)** basal width of seminal vesicles **(F)** discriminant analysis of basal width. MQD: drones from mated queens; VQD: drones from virgin queens; WBD: drones from worker bees.

We found that some indicators of the mucous glands of three groups of drones were at a stable level of variation right after rapid development from 0 to 4 days, including length, end width, middle width, and basal width. (Figures 2A–D). The length of the seminal vesicles slowly decreased after 4 days, while the middle width showed an increasing trend throughout the developmental cycle with increasing age. The basal width of the seminal vesicles also remained at a stable developmental level after a rapid developmental period from 0 to 4 days. Significant differences were also observed in the discriminant analysis of the indicators (Figures 3A–F). There were some overall differences in gonadal indicators among the different drones, which were not significant (*p* > 0.05).

### 3.3 Sperm counts

**FIGURE 4:**
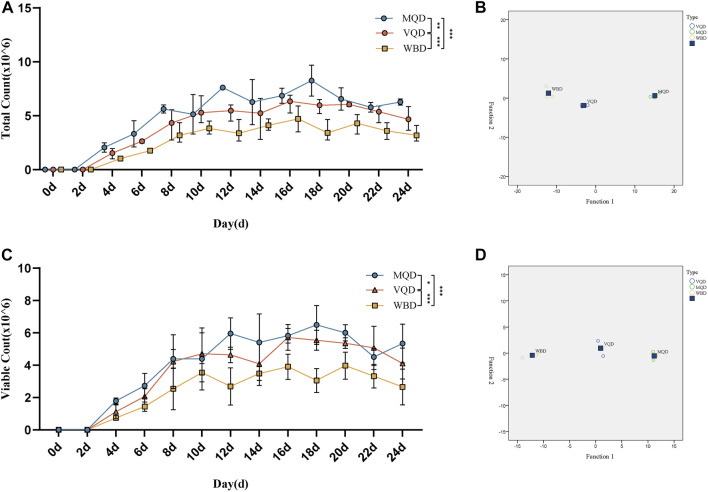
Developmental changes in total sperm count and sperm viability with age in drones: **(A)** total sperm count; **(B)** discriminant analysis of sperm count; **(C)** sperm viability; **(D)** discriminant analysis of sperm viability. MQD: drones from mated queens; VQD: drones from virgin queens; WBD: drones from worker bees.

There was rapid growth in total sperm count and sperm motility in drones from 0 to 18 days and a slow decrease after 18 days (Figures 4A, C). There were significant differences in the developmental course of the three groups of drones (Figures 4B, D).

### 3.4 Correlation analysis

**FIGURE 5:**
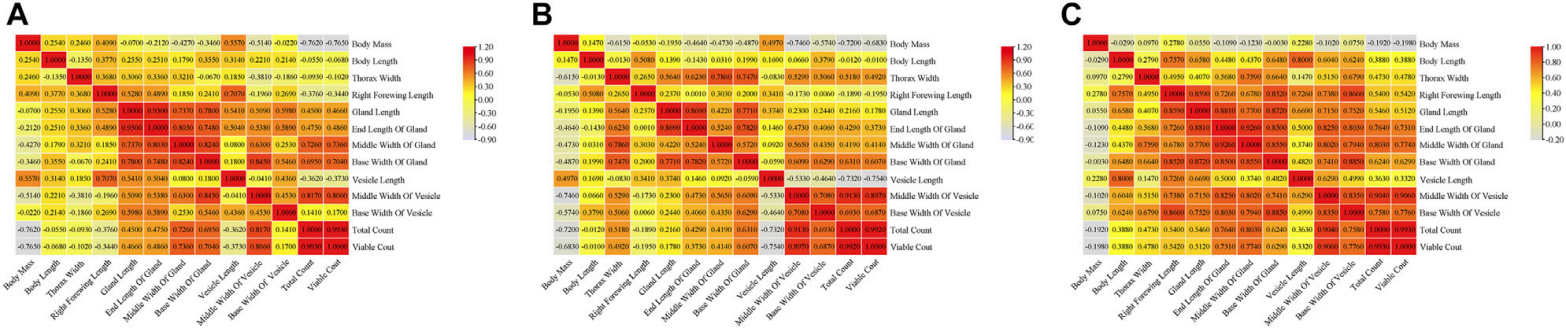
Correlation analysis of indicators in three groups of drones:**(A)** correlation matrix among indicators of MQD; **(B)** correlation matrix among indicators of VQD; **(C)** correlation matrix among indicators of WBD.

Correlation analyses were carried out for indicators of the three groups of drones. The results showed there were similar correlations among the three groups of drones. Significant positive correlations were found in morphological indicators, such as body mass, body length, thorax width, and right forewing length in MQD. There were also significant positive correlations among mucous gland indicators, seminal vesicle indicators, and sperm indicators, except for seminal vesicle length. This negative correlation was confirmed by the fact that the length of seminal vesicles decreases slowly throughout development while increasing sperm count leads to an increase in sperm width. In addition to body mass, significant relevance was found to indicators between the gonads and other morphologies. We suspected that the pre-flight preparation of drones, which led to a decrease in body weight, was responsible for this negative correlation. It would seem that body size is not the main determinant of sperm count (Figure 5A), as sperm count was negatively correlated with body mass, body length, thorax width, and right forewing length. The developmental trend of the seminal vesicles was also consistent with its negative correlation. Except for body length, there was a negative correlation between body mass and all other indicators, as well as in MQD (Figure 5A). Significant positive correlations among the morphological indicators, mucous gland indicators, seminal vesicle indicators, and sperm viability were found in the WBD, except for body mass. This correlation was particularly evident in WBD. It was also observed for the first time in WBD that seminal vesicle length was significantly correlated with seminal vesicle width and sperm count. We suspected that was some variation in WBD to improve their reproductive success. In three groups of drones, body mass showed negative correlations with other indicators, and there were only positive correlations among seminal vesicles length, seminal vesicles width, and sperm count in WBD, which were not in the other drones. This showed that seminal vesicle development was closely related to sperm viability.

### 3.5 GC-MS analysis

**FIGURE 6:**
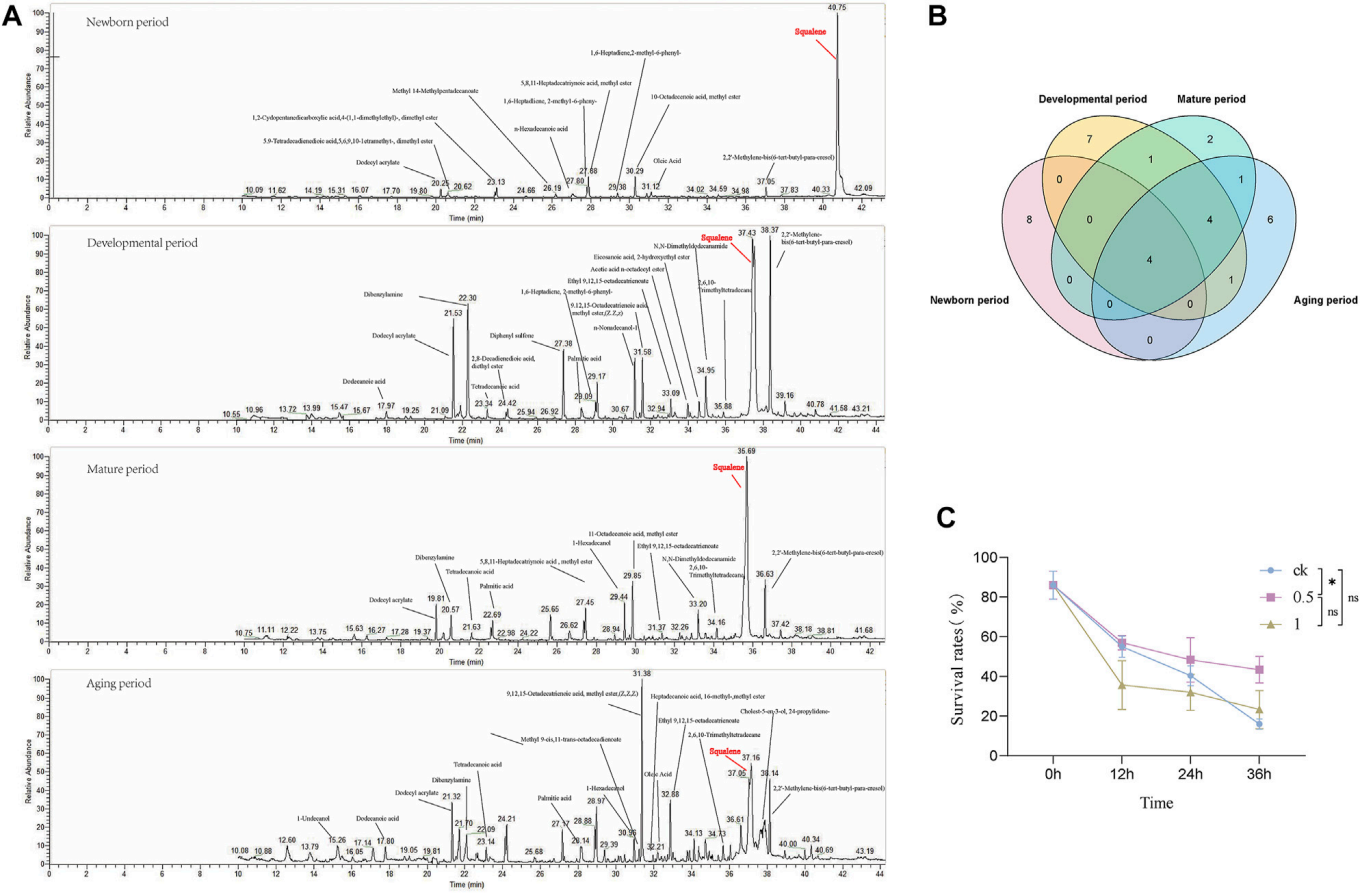
The developmental stages of MQD seminal vesicle by GC-MS analysis and *in vitro* validation experiments. **(A)** Newborn period, Developmental period, Mature period, and Aging period **(B)** Venn diagram of the distribution of metabolic substances at various periods of seminal vesicle development in MQD.**(C)** Validating Squalene *in vitro*.

To explore the metabolite basis within the seminal vesicles related to sperm viability, GC-MS analysis was used for MQD seminal vesicles. The development of the seminal vesicles was defined as four periods: newborn period (0 d), developmental period (4 d), mature period (12 d), and aging period (24 d). The metabolite of the seminal vesicle is very different during the four periods by GC-MS analysis. The highest content of squalene was found in the newborn period, followed by dodecyl acrylate, oleic Acid, 10-Octadecenoic acid, methyl ester, and 5,8,11-Heptadecatriynoic acid, methyl ester (Figure 6A). Squalene still shows high levels in the mature period, and many unique substances appear during this period, such as myristic acid, and stearyl acetate (Figure 6A). The level of squalene decreases significantly in the aging period, as well as sperm viability. There was a similar trend of levels in squalene and sperm viability from the newborn period to the aging period. So we suspect that squalene may be important to the sperm in the seminal vesicles, and it greatly maintains sperm activity. Four metabolites were identified at each developmental period: dodecyl acrylate, palmitic acid, 2,2′-Methylene-bis(6-tert-butyl-para-cresol), and squalene (Figure 6B). The levels of the metabolites wound change in the developmental process with the development of sperm vesicles and sperm viability. This could serve as a potential target substance for us to explore differences in sperm viability.

### 3.6 *In vitro* bioassay

From the four substances identified in the four periods, we selected squalene for *in vitro* bioassay. Different concentrations of squalene had a protective effect on sperm viability *in vitro* sperm storage assay. Among them, 0.5 mol/L squalene has been verified to be the most effective for storage, with a significant difference from the control group at 36 h (*p* < 0.05) (Figure 6C). It did not seem to have a protective effect on sperm viability, which a concentration was 1 mol/L in the experiment (*p* > 0.05).

## 4 Discussion

Poor environmental conditions caused a direct cause of developmental deformities and reduced fecundity in drones during the developmental process, including changes in temperature, poor nutrition, and chemical abuse (Winston, 1991; Bieńkowska et al., 2011; Czekońska et al., 2013; Stürup et al., 2013). Lower-quality sperm was used by the virgin queens to multiply, which affected the reproduction of colonies (Pettis et al., 2016; Rousseau and Giovenazzo, 2016; Straub et al., 2016; Kulhanek et al., 2017). There were few studies on the effects of drone sperm motility and seminal vesicle development on virgin queens insemination and population reproduction.

In our study, there was a similar trend throughout the development of three groups of drones. There was a clear dominance of MQD, including body mass, body length, thorax width, and right forewing length, which was consistent with the findings of Berg *et al.* Significant differences were found in the mucous glands and seminal vesicles throughout the development, which was the main reproductive glands of drones. The results suggested that MQD has a definite advantage at the level of reproductive development, and led to a strong competitive position during natural mating (Berg et al., 1997).

Sperm viability is an important indicator of the reproductive potential of drones. No sperm was found in the seminal vesicles of drones that had just fledged in *Apis mellifera* (Metz and Tarpy, 2019). In our study, the sperm was found in drones at 4 d. There was a peak in sperm counts about 18 days, after which it gradually declined. Sperm concentration increased, while semen volume decreased, followed by the color of semen changing from milky to creamy (Metz and Tarpy, 2019). Semen volume was not explored in the study, which merits further validation and comparison in the future.

Sperm was transferred from the testes to the seminal vesicles after plumage in drones, where they develop and mature, and in preparation for reproduction with virgin queens (Hayashi and Satoh, 2019). The metabolite levels of the seminal vesicle were related to sperm viability, which was the primary reproductive gland for storing sperm. The variations of lipids have been demonstrated in the seminal vesicles of mated queens in *Apis mellifera*, including fatty acyl and glycerol phospholipids (GPL), acrolein and sterol lipids (Liu et al., 2020). It is as expected, these substances were also found in the seminal vesicles of drones, including dodecyl acrylate, ethyl 9,12,15-octadecatrienoate, and palmitic acid. It was the first time that squalene was found in the seminal vesicles of drones. It has been shown that squalene plays a significant role in many aspects, including anti-oxidation, prevention of cell mutation, and liver protection (Bidooki et al., 2022; Shafaq et al., 2022; Zhang et al., 2022). Thus, we speculate that squalene maintains sperm activity in the hypoxic environment of the seminal vesicles, which was demonstrated *in vitro* semen storage experiments. There was no significant protective effect of three metabolites in sperm viability, including 2′-Methylene-bis (6-tert-butyl-para-cresol), dodecyl acrylate, and palmitic acid, and we consider that result of low levels and insignificant changes of three metabolites throughout the development of drones. We would verify the protective effect of these metabolites in the future.

We found a negative correlation among seminal vesicle length, seminal vesicle width, and sperm count in the development of MQD and VQD, as opposed to WBD. We consider the changes that occurred in the seminal vesicle of WBD, which improves its reproductive success in WBD. Sperm count was found to be negatively correlated with seminal vesicle length and significantly positively correlated with seminal vesicle width in the correlation analysis. We found that the length of the seminal vesicles decreased during the development process, while the number of sperm increased, resulting in the vesicles needing more space to store sperm. In addition, we found that the width of the seminal vesicles increased slowly throughout the developmental process. We suspect that this may be a feature in the reproductive development of drones during sexual maturation. This is the first time that we have found a slow decrease in seminal vesicle length and an increase in width during the development of drones in *Apis cerana*.

The developmental process has been described in drones, including the testis-seminal vesicle developmental system established from the larval stage to the adult stage together with the determination of the mating and insemination abilities of drones after adult plumage (Metz and Tarpy, 2019; Lago et al.,2020; Klein et al.,2021). It has been used to assess the reproductive potential of drones. The differences in metabolite content of seminal vesicles among drones can be used to assess the reproductive potential of drones. It is possible to use it as a basis for investigating the differences in reproductive potential and understanding the mechanisms behind these differences in drones.

## 5 Conclusion

The first comparison of spermatophore developmental levels in different drones, as well as an analysis of spermatophore metabolites at each developmental stage, was carried out in our study. It was found that squalene may act as a key substance in spermatophore to maintain sperm viability, which gives a new direction to study the differences in sperm viability among drones.

## Data Availability

The original contributions presented in the study are included in the article/Supplementary Material, further inquiries can be directed to the corresponding author.
