# Supplementary material for: Spermatophore development in drones indicates the metabolite support for sperm storage in honey bees (Apis cerana)
Source: Front Physiol. 2023 Feb 22;14:1107660. doi: 10.3389/fphys.2023.1107660 (PMC9992413; doi:10.3389/fphys.2023.1107660)
Supplement: Supplementary file 1 [file DataSheet1.PDF]

Supplementary information

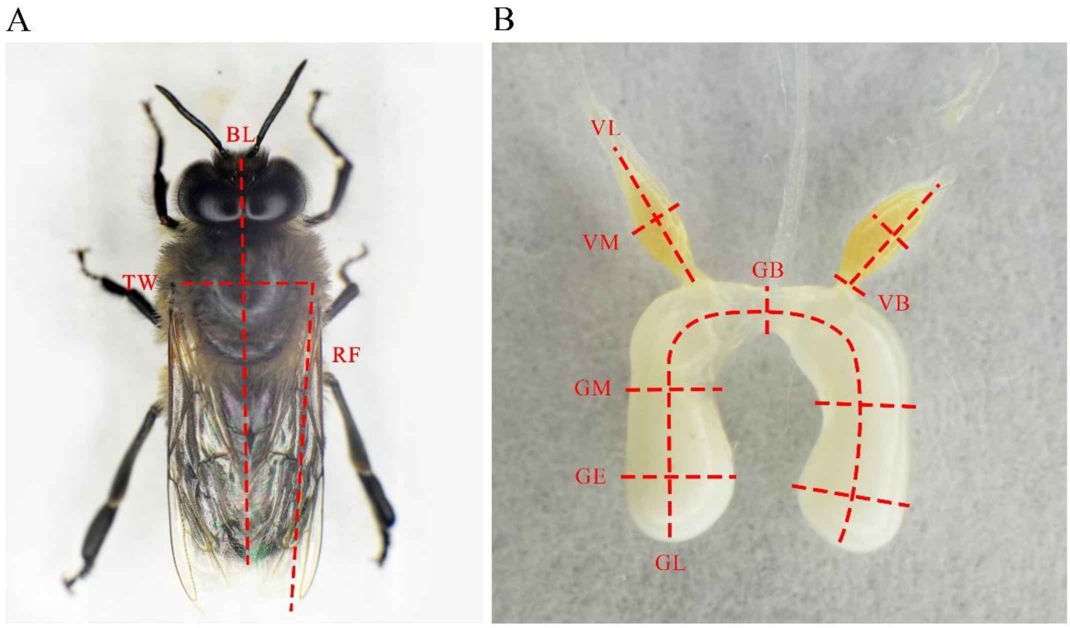

Supplementary Figure 1:A: Morphological indicators. TW:Throax Width BL: Body Length RF:Right Forewing Length B: Reproductive indicators. GL:Gland Length GE:End Length Of Gland GM: Middle Width Of Gland GB: Basal Width Of Gland VL: Vesicle Length VM: Middle Width Of Vesicle VB:Basal Width Of Vesicle

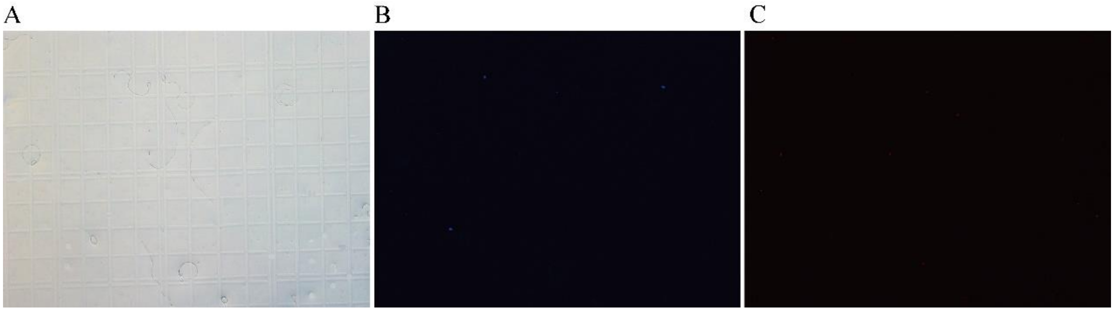

Supplementary Figure 2: Diagram of sperm staining. A. Sperm under the white light B. Living Cells C. Dead cells
